# Microbiological characterization of *Streptococcus pneumoniae* and non-typeable *Haemophilus influenzae* isolates as primary causes of acute otitis media in Bulgarian children before the introduction of conjugate vaccines

**DOI:** 10.1186/1476-0711-12-6

**Published:** 2013-03-25

**Authors:** Lena P Setchanova, Tomislav Kostyanev, Alexandra B Alexandrova, Ivan G Mitov, Dimitar Nashev, Todor Kantardjiev

**Affiliations:** 1 Department of Medical Microbiology, Medical University of Sofia, Faculty of Medicine, Sofia, 1431, BULGARIA; 2 Department of Microbiology, National Center of Infectious and Parasitic Diseases, Sofia, 1504, BULGARIA

**Keywords:** AOM, *S. pneumoniae*, *H. influenzae*, Serotypes, Antibiotic resistance

## Abstract

**Background:**

Pneumococcal and *Haemophilus influenzae* type b (Hib) vaccines were introduced in our national immunisation program in April 2010. The aims of this retrospective, laboratory-based study were to determine the serotypes and antibiotic resistance of *Streptococcus pneumoniae* and *H*. *influenzae* isolates from middle ear fluid (MEF) collected before the introduction of immunization.

**Methods:**

*S. pneumoniae* (n = 128) and *H. influenzae* (n = 40) strains isolated from MEF of children with AOM between 1994 and 2011 were studied. MICs were determined by a microdilution assay. Serotyping of *S. pneumoniae* was done by Quellung method and PCR capsular typing was used for *H. influenzae*. Macrolide resistance genes were detected by PCR for erythromycin resistant *S. pneumoniae* (ERSP). DNA sequencing of *fts*I gene was performed for ampicillin nonsusceptible *H. influenzae*.

**Results:**

The most common serotypes found among children with pneumococcal AOM were 19 F (20.3%), 6B (15.6%), and 19A (10.9%). The potential coverage rates by the PCV7, PCV10 and PCV13 of children aged < 5 years were 63.6%, 66.4% and 85.5%, respectively. Reduced susceptibility to oral penicillin was seen in 68.1%; resistance to erythromycin was 46.9%. We found *erm*(B) gene in 56.7% of the ERSP, *mef*(E) gene in 25%; 15% harbored both genes *erm*(B) + *mef*(E) and 3.3% had mutations of L4 ribosomal protein. Of the 40 *H. influenzae* isolates 97.5% were nontypeable. Nonsusceptibility to ampicillin occurred in 25%. Ampicillin resistance groups were: β-lactamase-positive ampicillin resistant (BLPAR) strains (10%), β-lactamase-negative ampicillin resistant (BLNAR) strains (12.5%) and β-lactamase-positive amoxicillin-clavulanate resistant (BLPACR) strains (2.5%). Among BLNAR and BLPACR most of the isolates (5/6) belonged to group II, defined by the Asn526Lys substitution.

**Conclusions:**

The levels of antibiotic resistance among *S. pneumoniae* and *H. influenzae* causing severe AOM in children are high in our settings. The existence of multidrug-resistant *S. pneumoniae* serotype 19A is of particular concern. The rate of BLNAR and BLPACR strains among *H. influenzae* isolates was 15%.

## Background

Acute otitis media (AOM) is the most common infection in children and the most common reason for which antibiotics are prescribed in this age group [1]. Currently, *Streptococcus pneumoniae* along with nontypeable *Haemophilus influenzae* are the major pathogens of AOM cases [2,3]. In a study of bacterial etiology of AOM between 2009 and 2010 in Bulgaria, the most frequently isolated pathogen was *S. pneumoniae* (47.6%), followed by *H. influenzae* (27.6%) [4].

Among the 93 pneumococcal serotypes, only a few are responsible for most cases of AOM in children worldwide, including serotypes 3, 6A, 6B, 9V, 14, 19A, 19F, and 23F [5,6]. Three pneumococcal conjugate vaccines (PCVs) are currently licensed for use in children and they have showed modest efficacy against AOM overall [7]. The 7-valent PCV was widely used throughout the world in the past 10 years. Recently, two new PCVs were introduced since 2010, PCV10 (Synflorix; GSK, UK) and PCV13 (Prevenar 13; Pfizer, USA). In children aged < 5 years with AOM, the PCV7, PCV10 and PCV13 covered 54%, 60% and 85% of the pneumococcal serotypes, respectively in Germany [8].

Vaccination against pneumococcal diseases with PCV10 was introduced in Bulgaria in our national immunization program (NIP) for the first time in April, 2010. PCV7 was not used in Bulgaria prior to PCV10 introduction. Additionally, *H. influenzae* type b (Hib) conjugate vaccine was also included for routine immunization of infants as part of a pentavalent vaccine since 1 st April, 2010. Both conjugate vaccines are free of charge.

The prevalence of antibiotic-resistant *S. pneumoniae* has increased globally before the pre-vaccine era. The pattern of changes in antimicrobial susceptibility varies among serotypes and geographic regions [9]. Prior to PCV7 introduction, the major problem with antimicrobial resistance in *S. pneumoniae* worldwide was non-susceptibility to penicillin, macrolides and multidrug resistance [10].

Resistance to β-lactams in *H. influenzae*, especially to ampicillin/amoxicillin, ranging from 3 to 65% in some countries is an increasing problem [11]. Although, ampicillin resistance is predominantly mediated by the β-lactamase production, alterations in penicillin-binding proteins (PBPs), mainly PBP 3, due to *ftsI* amino acid substitutions occur in some parts of the world [12]. In addition, β-lactamase positive, amoxicillin-clavulanate-resistant strains have been described due to the combination of both mechanisms [13].

The objectives of our study were to determine serotypes and antibiotic susceptibility of *S. pneumoniae* and *H. influenzae* isolates from middle ear fluid (MEF) cultures collected before the introduction of conjugate vaccines in the NIP. The presence of macrolide resistance determinants in *S. pneumoniae* isolates and the prevalence of mechanisms of ampicillin resistance in *H. influenzae* strains were also studied.

## Methods

### Patients and isolates

Pediartic (< 14 years of age) *S. pneumoniae* (n = 128) and *H. influenzae* (n = 40) isolates from middle ear fluid cultures obtained from children with the clinical diagnosis “acute otitis” complicated with otorrhea were examined. The AOM episodes were confirmed by an otorrinolaryngologist or pediatrician. The strains were isolated from several microbiological laboratories throughout Bulgaria between 1994 and 2011 and isolates had been sent in our department on a voluntary basis through a passive, laboratory-based study for retrospective analysis. Only one episode of AOM or one isolate of any of the two pathogens was included per patient, except for one child from whom both were detected from the same sample (mixed infection). Serotypes of *S. pneumoniae* were distributed per age group (≤ 2 years, 2–5 years, and >5-14 years); 90% of *H. influenzae* strains were isolated from children aged less than 5 years. None of the children with AOM had been immunized with pneumococcal or Hib conjugate vaccines. Five pneumococcal strains isolated in 2011 were also included because they were from children which had not received vaccines.

*S. pneumoniae* strains were confirmed with both methods - optochin susceptibility test and bile solubility. *H. influenzae* isolates were identified by Gram-stain morphology, growth requirements for factor V, X, and porphyrin (A.L.A. disks, Remel, Lenexa, Kansas, USA), biochemical tests according to standard methods and the presence of *iga* protease gene determined by conventional PCR [14,15].

### Antimicrobial susceptibility test

The antibiotic susceptibilities were determined by the broth microdilution method according to guidelines of Clinical and Laboratory Standards Institute (CLSI) [16]. Minimal inhibitory concentrations (MICs) were performed on Microtiter plates (Sensititre, Trek Diagnostic Systems Ltd., UK). STR6F MIC plate was inoculated with cation-adjusted Mueller-Hinton broth and 5% lysed horse blood for *S. pneumoniae*, and for *H. influenzae* HPB-*Haemophilus/Streptococcus pneumoniae* plate was inoculated with *Haemophilus* test broth. Final bacterial density was 5 × 10^5^ CFU/ml and the plates were incubated at 35°C for 20–24 h in 5% CO_2_. The MICs were interpreted according to CLSI, 2010 [16]. All *H. influenzae* strains were tested for β-lactamase production by the chromogenic cephalosporin test with nitrocefin as substrate. Standard quality control strains were *S. pneumoniae* ATCC 49619 and *H. influenzae* ATCC 49247. Strains resistant to ≥ 3 classes of antibiotics were defined as multidrug resistant (MDR).

### Serotyping

Serogrouping of *S. pneumoniae* was performed using the latex agglutination method (Pneumotest-Latex kit, Statens Serum Institut, Copenhagen, Denmark). Serotyping was performed later with the capsular Neufeld Quellung method by using some common factor antisera (SSI, Copenhagen, Denmark) [17]. *H. influenzae* strains were serotyped by a PCR capsule typing method, detecting the capsule-producing gene - *bexA*. The algorithm used for detection of the *cap* genes and for determining capsular serotypes was that proposed by Falla et al. [18].

### Detection of macrolide resistance determinants in *S. pneumoniae*

The presence of *erm*(B) and *mef*(A) was evaluated concurrently in a duplex reaction by PCR for all erythromycin-resistant *S. pneumoniae* (ERSP) isolates. The primer sets were as described by Sutcliffe et al. [19]. Gene amplification was performed using a Techgene-thermal cycler (Techne, England). *Mef*(A) and *mef*(E) were then distinguished by PCR restriction fragment length polymorphism analysis of the 348-bp *mef* amplicon with BamHI restriction enzyme (New England Biolabs), which has no restriction site in *mef*(E) and one in *mef*(A), generating two fragments of 284 and 64 bp [20]. Mutations in ribosomal protein L4 were determined as described elsewhere [21].

### DNA sequencing analysis of *ftsI* gene in *H. influenzae*

The DNA fragment encoding the transpeptidase region of PBP 3 was amplified by PCR from strains which were nonsusceptible to ampicillin (MICs of ≥ 2 μg/ml). Amplification of *ftsI* gene was done using primers and PCR conditions described previously [22]. Nucleotide and amino acid sequences were analysed using DNAMAN 4.11 Software (Lynnon BioSoft, Canada). Deduced amino acid sequences were compared with the reference sequence obtained from the *H. influenzae* Rd strain. Strain ATCC 49247 was included as an *H. influenzae* BLNAR control strain.

We classified *H. influenzae* strains according to their ampicillin susceptibility, β-lactamase production, and on the basis of the DNA sequencing results obtained from ampicillin-nonsusceptible isolates. Four main classes were determined: β-lactamase negative, ampicillin-susceptible strains with MIC of ≤ 1 μg/ml (BLNAS); strains producing β-lactamase, and without mutations in the *ftsI* gene (BLPAR); β-lactamase negative, ampicillin-nonsusceptible strains (MICs of ≥ 2 μg/ml) which have amino acid substitutions in the *ftsI* gene (BLNAR); and isolates that have both mechanisms (β-lactamase production and amino acid substitutions in the *ftsI* gene)(BLPACR).

## Results

### Serotype distribution among *S. pneumoniae* and *H. influenzae*

Serotypes of 128 *S. pneumoniae* isolates obtained from MEF are presented in Table 1. The strains are divided in three age groups: children aged < 2 years (50.0%), children aged >2 to 5 years (35.9%), and children aged > 5 y - 14 y (14.1%). The leading serotype among all children with pneumococcal AOM was 19 F (20.3%), followed by serotypes 6B (15.6%), 19A (10.9%), serotypes 14 and 9 V (8.6% each), and 23 F (7.8%). Serotypes 19A, 3 and 6A included in the PCV13 accounting altogether 18.7% among pneumococcal strains.

**Table 1:** **Serotype distribution of *S. pneumoniae *isolates from children with acute otitis media by age group and potential vaccine coverige: 1994-2011**

**Serotype**	**No. of isolates per age group**				**Total no. (%) of particular serotype**
	**≤ 2 y**	**> 2–5 y**	**≤ 5 y**	**> 5 y - 14 y**	
**PCV7 serotypes**					
19 F	17	7	**24**	2	26 (20.3)
6 B	8	9	**17**	3	20 (15.6)
14	10	-	**10**	1	11 (8.6)
9 V	5	3	**8**	3	11 (8.6)
23 F	5	4	**9**	1	10 (7.8)
18 C	1	1	**2**	1	3 (2.3)
Total no.	46	24	70	11	81 (63.3)
**Other vaccine serotypes**					
19 A	9	4	**13**	1	14 (10.9)
3	2	3	**5**	1	6 (4.7)
6 A	1	2	**3**	1	4 (3.1)
7 F	1	1	**2**	-	2 (1.6)
1	-	1	**1**	1	2 (1.6)
Total no.	13	11	**24**	4	28 (21.9)
**Non-vaccine serotypes**					
15 B	2	3	**5**	-	5 (3.9)
23 A	2	1	**3**	-	3 (2.3)
11^a^	-	2	**2**	-	2 (1.6)
13,28^b^	-	1	**1**	1	2 (1.6)
15 C	-	2	**2**	-	2 (1.6)
10^a^	1	1	**2**	-	2 (1.6)
16/36/37^b^	-	-	**-**	1	1 (0.8)
24/31/40^b^	-	1	**1**	-	1 (0.8)
NT^c^	-	-	**-**	1	1 (0.8)
Total no.	5	11	**16**	3	19 (14.8)
**Overall no. (%)**	64 (50.0)	46 (35.9)	**110 (85.9)**	18 (14.1)	128 (100.0)
**Percentage coverage rate of pneumococcal conjugate vaccines**					
PCV7	71.9	52.2	**63.6**	61.1	63.3
PCV10	73.4	56.5	**66.4**	66.7	66.4
PCV13	92.2	76.1	**85.5**	83.3	85.2

^a^ The strains were determined to serogroup.

^b^ Expected to be serotypes/serogroups (Strains were positive only for one of the pooled sera).

^c^ Non-typeable strains with Pneumotest-kit sera.

Coverage by serotypes included in the current PCV was evaluated and the calculations were performed without accounting for possible cross-protection. The potential coverage rates by the PCV7, PCV10 and PCV13 for children aged < 5 years were 63.6%, 66.4% and 85.5%, respectively.

Forty MEF isolates from children with AOM were identified as *H. influenzae*. Nontypeable *H. influenzae* (NTHi) strains predominated (n = 39; 97.5%), and one was encapsulated serotype a isolate (2.5%).

### Antimicrobial susceptibility

Antibiotic susceptibility data for 128 *S. pneumoniae* isolates are presented in Table 2. Reduced susceptibility to penicillin (oral penicillin V) was seen in 68.1% of the strains. This included strains (28.1%) with high level of resistance to penicillin (MIC ≥ 2 mg/L). The rate of resistance to macrolides (erythromycin) was 46.9%. Susceptibility results to amoxicillin shows 6.2% intermediately resistant strains and 4.7% fully resistant isolates. In a pre-vaccine era the percentage of multidrug-resistant strains was 53.9% (69/128). In order to observe any changes in antibiotic resistance over the time, all pneumococcal strains were divided in two periods depending on the year when the strain was isolated: 1994–2005 and the more representative pre-vaccine period 2006–2011 (Table 2). During the second period we revealed increased resistance to erythromycin from 36.7% to 53.2%, and to clindamycin from 22.4% to 40.5%, respectively. Non-susceptibility rates to amoxicillin, cefuroxime sodium and ceftriaxone were found in 8.2%, 28.6% and 4.1%, respectively in the first period, and were increased up to 12.7%, 41.8% and 8.9%, respectively in the second period 2006–2011.

**Table 2:** **Antimicrobial susceptibility of 128** ***S. pneumoniae *isolates from middle ear fluids and trends in antibiotic nonsusceptibility divided in two periods depending on year of strain isolation**

**Antibiotics**	**MICs (μg/mL)**		**Overall% susceptibility n = 128**			**NS rates in 1994–2005 n = 49**	**NS rates in 2006-2011n = 79**
	**MIC**_**50/90**_	**MIC range**	**S**	**I**	**R**	**%**	**%**
Penicillin	0.12/4.0	0.01 - 8.0	31.2	40.6	28.1	73.5	65.8
Amoxicillin	0.12/4.0	0.01 - 8.0	89.1	6.2	4.7	8.2	12.7
Cefuroxime sodium	≤0.5/8.0	≤0.5- ≥ 16.0	63.3	5.5	31.2	28.6	41.8
Ceftriaxone	0.06/1.0	0.03-4.0	93.0	4.7	2.3	4.1	8.9
Erythromycin	≤0.25/≥64.0	≤0.25- ≥ 64.0	53.1	0.0	46.9	36.7	53.2
Clindamycin	0.06/≥64.0	0.01- ≥ 64.0	66.4	0.8	32.8	22.4	40.5
Levofloxacin	1.0/1.0	0.5 - 2.0	100.0	0.0	0.0	0.0	0.0
Tetracycline	2.0/≥8.0	0.25- ≥ 8.0	50.8	0.8	48.4	46.9	50.6
Chloramphenicol	4.0/≥8.0	0.5- ≥ 8.0	84.4	-	15.6	24.5	10.1
Trimeth/Sulfameth	2.0/≥4.0	0.06- ≥ 4.0	35.2	22.7	42.2	65.3	64.6

*MICs*-minimal inhibitory concentracions; *MIC*_*50/90*_, MICs for 50% and 90% of the isolates, respectively; *R*-resistant; *I*-intermediate; *S*-susceptible (Interpretation according to CLSI, 2010); *NS***-** nonsusceptibility; The following breakpoints (μg/mL) for penicillin (oral penicillin V) were used: susceptible ≤ 0.06; intermediate ≥ 0.12 - 1.0; resistant ≥ 2; Trimethoprim/Sulfamethoxazole (1:19 ratio), MIC refers to trimethoprim value.

A total of 40 *H. influenzae* strains were tested for susceptibility to antibiotics. High-nonsusceptibility rates were found to trimethoprim-sulfamethoxazole - 35.0% (14/40), and to ampicillin - 25.0% (10/40). Of the 10 ampicillin nonsusceptible *H. influenzae* strains, 9 were fully resistant to ampicillin (MIC ≥ 4 mg/L) and one was intermediately susceptible (MIC = 2 mg/L) isolate (Table 3). Ceftriaxone and levofloxacin were the most active agents tested, and all isolates were fully susceptible to these agents.

**Table 3:** **Distribution of ampicillin resistance classes of paediatric middle ear fluids *H. influenzae *isolates according to their β-lactamase production, susceptibilities to ampicillin and amoxiclav, and amino acid substitutions present in ampicillin nonsusceptible strains with mutations in *ftsI *gene**

**Resistance class**	**No. of strains (%)**	**β-lac**	**MIC (mg/L)**		**Amino acid substitution(s) in *ftsI *gene**	**Group**
			**Ampicillin**	**Amoxiclav**		
BLNAS	30 (75.0)	-	≤ 1.0	≤ 2.0	ND	-
BLPAR	4 (10.0)	+	≥ 8.0	≤ 2.0	No changes	-
BLNAR	1 (2.5)	-	4.0	4.0	Arg517His	I
BLNAR	1 (2.5)	-	2.0	4.0	Asn526Lys	IIa
BLPACR	1 (2.5)	+	≥ 8.0	4.0	Asp350Asn, Gly490Glu, Asn526Lys, Ala530Ser	IIa
BLNAR	2 (5.0)	-	4.0	8.0	Asp350Asn, Met377Ile, Gly490Glu, Ala502Val, and Asn526Lys	IIb
BLNAR	1 (2.5)	-	4.0	8.0	Asp350Asn, Ala502Thr, Asn526Lys	IIc
Total	40 (100.0)					

Abbreviations: *β-lac*, β-lactamase production; *Amoxiclav*, amoxicillin-clavulanic acid (2:1 ratio). *MIC* refers to amoxicillin value. *ND*, DNA sequencing of *ftsI* gene was not done; *BLNAS*, β-lactamase negative, ampicillin-susceptible strains; *BLPAR*, β-lactamase positive ampicillin-resistant *H. influenzae* without amino acid substitutions in the PBP 3; *BLNAR*, β-lactamase negative ampicillin-resistant *H. influenzae* with amino acid substitutions in the PBP 3; *BLPACR*, β-lactamase positive strains with amino acid substitutions in the PBP 3; No changes along the section of amino acids 350 to 530. The strains with *ftsI* gene mutations were classified into groups according to the proposed criteria by Dabernat et al. [22].

### Macrolide resistance determinants among *S. pneumoniae* isolates

Of the 128 MEF isolates 60 (46.9%) were ERSP. The distribution of macrolide resistance determinants and particular serotypes are presented in Table 4. We found *erm*(B) gene in 34 isolates (56.7%), *mef*(E) gene in 15 isolates (25.0%), 9 isolates harbored both genes *erm*(B) + *mef*(E) (15.0%), and 2 (3.3%) strains had mutations of L4 ribosomal protein. The predominant serotypes among *erm*(B) genotype were 6B (n = 16), 19A (n = 9), and 19 F (n = 6). Of the 15 isolates harboring *mef*(E) gene the prevalent serotypes were 14 (n = 10) and 19 F (n = 3). The dual resistance mechanism *erm*(B) + *mef*(E) was found in 19 F (n = 7) and 19A (n = 2) serotypes. Of note, resistance mechanism L4 found in two strains isolated in 1995 has never been seen in the years after. The coverage rates of the PCV7, PCV10 and PCV13 for ERSP were 75.0%, 75.0% and 96.7%, respectively. Considering co-resistance rates to penicillin according to macrolide resistance genotype, the percentage of strains exhibiting non-susceptibility to oral penicillin (MIC ≥ 0.1 mg/L) was found in 93.3% of ERSP isolates overall. The proportion of fully resistant to penicillin ERSP isolates was 17.6% in strains harboring *erm*(B) gene, 20.0% in these carried *mef*(E) gene, whereas 66.7% of isolates showing the dual resistance mechanism *erm*(B) + *mef*(E) were fully resistant to oral penicillin.

**Table 4:** **Genotype distribution of 60 erythromycin-resistant *S. pneumoniae *(ERSP) isolates from middle ear fluids: association with serotypes and co-resistance to penicillin**

**Resistance genotype**	**Total no. (%) (n = 128)**	**Co-resistance to penicillin**^**1**^**n (%)**			**Serotypes of ERSP (n)**
		**R**	**I**	**S**	
*erm*(B)	34 (56.7)	6 (17.6)	25 (73.5)	3 (8.8)	6B (16); 19A (9); 19 F (6); 14, 23 F, 23A (1) each
*mef*(E)	15 (25.0)	3 (20.0)	11 (73.3)	1 (6.7)	14 (10); 19 F (3); 6B, 15B (1) each
*erm*(B) + *mef*(E)	9 (15.0)	6 (66.7)	3 (33.3)	-	19 F (7); 19A (2)
L_4_^2^	2 (3.3)	2 (100.0)	-	-	19A (2)
Total no. (%) of ERSP	60 (46.9)	17 (28.3)	39 (65.0)	4 (6.7)	-

*R*-resistant; *I*-intermediate; *S*-susceptible (Interpretation according to CLSI, 2010); ^1^The following breakpoints (μg/mL) for penicillin (oral penicillin V) were used: susceptible ≤ 0.06; intermediate ≥ 0.12 - 1.0; resistant ≥ 2. ^2^ Mutations in ribosomal protein L4 were determined as described elsewhere [21].

### Ampicillin-resistant mechanisms and amino acid substitutions of PBP 3 among MEF *H. influenzae* isolates

The total collection of 40 *H. influenzae* was grouped according to our definition of ampicillin resistance classes: BLNAS strains (n = 30; 75.0%), BLPAR (n = 4; 10.0%) isolates without mutations in the *ftsI* gene, BLNAR strains (n = 5; 12.5%) with amino acid substitutions in the *ftsI* gene, and BLPACR (n = 1; 2.5%) strain that was β-lactamase positive, and had mutations in the critical region of *ftsI* gene with amino acid substitutions in PBP 3. Table 3 shows these ampicillin-resistance classes according to their β-lactamase production, susceptibilities to ampicillin and amoxicillin-clavulanic acid, and amino acid substitutions which present in ampicillin nonsusceptible strains. Of note, among six strains with amino acid substitutions in PBP 3 (5 BLNAR and one BLPACR), three were resistant to amoxicillin-clavulanic acid, and three strains were susceptible according to CLSI breakpoints.

The nucleotide sequences of the *ftsI* gene were determined for 10 ampicillin-nonsusceptible *H. influenzae* strains (MICs ≥ 2 μg/ml) and their deduced amino acid sequences were compared with those of the ampicillin-susceptible Rd strain. Five β-lactamase nonproducing strains had significant amino acid substitutions in the *ftsI* gene, thereby we categorizing them as BLNAR strains. Among remaining 5 β-lactamase positive strains, one isolate had amino acid substitutions in PBP 3. According to the classification of Dabernat et al. [22] different mutation patterns were identified among BLNAR and BLPACR strains. The Arg517His substitution that defines group I was observed in one ampicillin nonsusceptible *H. influenzae*. Most isolates (n = 5) belonged to group II, defined by the Asn526Lys substitution, and could be further subdivided into three subgroups - IIa, IIb and IIc. Group III mutations in *ftsI* gene conferring high-level resistance were not detected.

## Discussion

This is a retrospective study about serotype distribution and antibiotic resistance of MEF *S. pneumoniae* and *H*. *influenzae* isolates collected from children before introduction of PCV10 and Hib conjugate vaccines nationwide. Microbiological diagnosis of AOM is not done on a routine basis in Bulgaria and this mild disease is underrepresented in our data set. Also, the tympanocentesis is not a common practice in our country. Thus, bacterial cultures studied here most likely represent cases of severe otitus media or patients with a complicated AOM.

The analysis included 128 *S. pneumoniae* isolates, of which 86% occurring in children aged ≤ 5 years. We found a relatively high rate of serotype 19F infections (20.3%), which is in accordance with the serotype distribution observed in the studies of AOM in German children [8], and the Finland [7]. Serotypes 6B and 19A were next most prominent, each comprising about 16% and 11%, respectively of the overall data set. The coverage calculations for the serotypes included in PCV7 without cross-protection was 63.6% in children with AOM aged < 5 years, which is almost the same as was the serotype coverage previously reported for invasive pneumococcal disease (IPD) for the same age group in Bulgaria [23]. The PCV10 increase the coverage by 2.8% of pneumococcal AOM (66.4%). The PCV13, which includes three serotypes in addition to the PCV10, would increase the coverage to 85.5%, mainly due to serotype 19A followed by serotypes 3 and 6A (Table 1). However the cross-protection of serotype 6B towards serotype 6A was not included in calculations for our study. We found 14 serotype 19A isolates, 13 of which were multidrug resistant.

The results showed a high rate of reduced oral penicillin nonsusceptibility (68%) and a very high rate of ERSP isolates (47%) among MEF strains. These rates are more than twice as high as previously recorded with IPD isolates in Bulgaria studied during the same year period [23]. These high resistance rates may be due to clonal spread of certain MDR pneumococcal serotypes such as 6B, 19 F and 19A. This resistance reflects the complexity of many of the severe otitis media episodes which included therapy failures. Notably, we observed that the percentages of macrolide, clindamycin, amoxicillin, cefuroxime sodium and ceftriaxone nonsusceptibility increased among *S. pneumoniae* strains isolated during the last 6 years of the study period. The high antibiotic consumption for the treatment of AOM represents the major pressure for resistance selection [24].

Overall, macrolide resistance in *S. pneumoniae* now is mainly due to *erm*(B) gene ( 71.7%), including 15% of isolates with a dual macrolide resistance mechanism (Table 4). This pattern of macrolide resistance determinants in the last years replaced the efflux mechanism of macrolide resistance (*mef* genotype), which was predominant among ERSP in Bulgaria before 2005 [25]. Macrolide-resistant *S. pneumoniae* strains were found to be co-resistant to oral penicillin (Table 4), and to other compounds, such as tetracycline and trimethoprim-sulfamethoxazole (data not shown). Thus, the prevalence of MLS_B_ genotype may be due to clonal spread of certain MDR pneumococcal serotypes as 6B, 19 F and 19A. Interestingly, we observed multi-resistant serotype 19A in many years ago. For the first time, this MDR serotype was found in 1995 with L4 mutations as the mechanism of macrolide resistance [21]. Since 2000 and after, *erm* (B) genotype was found in serotype 19A isolates (nine strains). Dual macrolide resistance mechanisms were observed recently, between 2008 and 2010 in two serotype 19A strains (Table 1 and Table 4). Both the emergence of a new clone or capsular switching might be associated with these changes in our 19A serotype isolates. A considerable increase in the disease caused by serotype 19A has been recorded after the introduction of PCV7 in some countries [24,26]. Of note, MDR serotype 19A isolates have also been identified in Israel and Finland before the large-scale use of conjugate vaccines as was the case in our study [27,28].

Our findings of 97.5% nontypeable *H. influenzae* strains isolated from AOM are in accordance with the literature [11,29]. Nontypeable *H. influenzae* are the major etiologic agents of respiratory tract infections in our country as was shown before [30]. Ampicillin/amoxicillin nonsusceptible *H. influenzae* were found to be a quarter of our MEF isolates. Among BLNAR and BLPACR most of the isolates (5/6) belonged to Dabernat group II, defined by the Asn526Lys substitution. A high incidence of BLNAR *H. influenzae* strains has been reported in Japan, Spain and France [11,12,22]. The emergence of BLNAR and BLPACR strains among MEF *H. influenzae* isolates may have implications for the treatment of AOM infections, because amoxicillin and amoxicillin-clavulanate are the most common antibiotics used in the treatment of AOM in our country.

The main limitations of our study are: 1) it covers a long period of time, and the power is low; 2) isolates were sent by microbiology labs in hospitals, and we do not have clinical information on the type of AOM; 3) as isolates came from hospitals, they are not representative of *S. pneumoniae* and *H. influenzae* isolates causing otitis in children attending primary medical centers; 4) the numbers of *S. pneumoniae* and *H. influenza*e strains are small, because only a portion of children with AOM in Bulgaria are tested. Despite these limitations, we believe that they may represent a rough estimate of the serotype distribution and antibiotic resistance among a group of children with complicated AOM. This information will be invaluable when planning vaccination strategies to protect against otitis media.

In conclusion, the levels of antibiotic resistance among *S. pneumoniae* and *H. influenzae* causing severe otitis media in children are high in our settings. This has important therapeutic implications because such antibiotic-resistant isolates compromise the choice of macrolides and amoxicillin as treatment of AOM episodes in children. The existence of MDR *S. pneumoniae* serotype 19A before the active pneumococcal immunization is of particular concern. The rate of 15% BLNAR and BLPACR strains among *H. influenzae* MEF isolates may have implications for the treatment of AOM. Ongoing surveillance is needed for both pathogens in order to observe any changes in serotypes prevalence and antibiotic resistance after introduction of conjugate vaccines in our country.

## Consent

Written informed consent was obtained from the patient’s guardian/parent/next in keen for publication of this report and any accompanying images

## Competing interests

All authors declare that they have no competing interests.

## Authors’ contributions

LS designed the study, participated in the laboratory analyses and experiments, and drafted the manuscript. TK and AA carried out technical help and participated in the experiments. IM monitored the progress and supervised the manuscript. DN and TK assisted in clinical isolates gathering. All authors read and approved the final manuscript.
